# A 17 year experience of attrition from care among HIV infected children in Nnewi South-East Nigeria

**DOI:** 10.1186/s12879-021-06099-3

**Published:** 2021-05-03

**Authors:** Chinyere Ukamaka Onubogu, Ebelechuku Francesca Ugochukwu

**Affiliations:** 1 Department of Paediatrics, Faculty of Medicine, Nnamdi Azikiwe University, Awka, Nigeria; 2 Paediatric Infectious Diseases Unit, Nnamdi Azikiwe University Teaching Hospital, Nnewi, Nigeria

**Keywords:** Childhood HIV, Retention in care, Death, Loss to follow-up, Sub-Saharan Africa

## Abstract

**Background:**

A large number of HIV-infected children continue to die despite reported scale-up of paediatric HIV services.

**Aim:**

The trend in attrition among children enrolled in an anti-retroviral therapy (ART) programme was evaluated.

**Methods:**

This was a retrospective review of children enrolled into NAUTH ART programme between 2003 and 2019.

**Results:**

1114 children < 15 years at enrolment were studied. The male: female ratio was 1:1 while median age at enrolment was 4.3 years. About two-thirds had WHO stage 3 or 4 disease at enrolment. The rate of loss to follow-up (LTFU) and death were 41.0 and 8.4%, respectively, with overall attrition incidence of 108/1000PY. Despite the downward trend, spikes occurred among those enrolled in 2008 to 2011 and in 2017. The trend in 6-, 12-, 24- and 36-months attrition varied similarly with overall rates being 20.4, 27.7, 34.3 and 37.3%, respectively. Among those on ART, > 50% of attrition was recorded within 6 months of care. Advanced WHO stage, young age, non-initiation on ART or period of enrolment (*P* <  0.001), and caregiver (*p* = 0.026) were associated with attrition in bivariate analysis. Apart from caregiver category, these factors remained significant in multivariate analysis. Most LTFU could not be reached on phone. Among those contacted, common reasons for being lost to follow-up were financial constraints, caregiver loss, claim to divine healing, family disharmony/child custody issues and relocation of family/child.

**Conclusion/recommendation:**

Attrition rate was high and was mostly due to LTFU. Predictors of attrition were late presentation, young age, delay in ART initiation and financial constraints. Efforts should be intensified at early diagnosis, linkage to care and implementation of “test and treat” strategy. Innovative child centered approaches should be adopted to enable the HIV-infected children remain in care despite challenges which can truncate treatment.

## Introduction

Infection with the human immunodeficiency virus (HIV) still presents a serious public health concern particularly among children. Despite efforts to improve services and outcomes of children living with HIV, a large number of children continue to die from HIV annually [1, 2]., Although children less than 15 years old constituted 4.5% of all individuals living with HIV in 2018, they accounted for 13% of the 770,000 deaths attributable to HIV [2]. In 2018, the incidence of HIV-related mortality in children tripled that of adults (5.9% versus 1.9%) [2].

More than 90% of HIV infection in children result from mother-to-child transmission (MTCT) during pregnancy, delivery or postnatally during breastfeeding [3]. Such perinatally acquired HIV is associated with more rapid progression due to immature immune system, and high mortality especially in sub-Saharan Africa [3]. Without any intervention, 50% of prenatally infected children die within their first two years of life. Fortunately, anti-retroviral therapy (ART) has enabled many children to grow into adulthood [3, 4]. However, the success of childhood HIV programmes depends on timely diagnosis, early initiation of treatment, adherence to medications and active follow-up, to ensure optimal outcomes. This is hinged on frequent visits to treatment sites for clinic consultations, laboratory monitoring and ARV drug refill. Due to changing life circumstances, care giver issues and other challenges, many children in developing countries may not meet up with the demands of life-long care and therefore unable to continue enjoying the life-saving benefits of ART. Consequently, the children are faced with high risk of treatment failure, death and HIV transmission [5].

In 2014, the Joint United Nations Programme on HIV/AIDS (UNAIDS) and partners set ambitious 90–90-90 fast-track targets, that 90% of people living with HIV know their status, 90% of people who know their status be on treatment, and 90% of people on treatment be virally suppressed by 2020 [6]. In order to end the AIDS epidemic, these targets were set at 95–95-95 by year 2030 [7]. However, these targets cannot be met if children fail to be retained in care. Children continue to lag behind adults across all cascades, and reports indicate that all paediatric targets set for 2016 were missed [1, 7]. Despite reported scale-up of testing and enrolment services, only about half of HIV-infected children less than 15 years were receiving ART in 2019 [7]. Besides, the confinement measures, restrictions on movement and economic stresses brought by the Covid-19 pandemic may consequentially cause disruption of services and treatment interruptions.

This study was carried out as a consequence, to determine the trend in attrition among children enrolled into the paediatric ART programme in Nnamdi Azikiwe University Teaching Hospital (NAUTH) Nnewi, Nigeria. It is hoped that the findings will provide the needed evidence to drive the adoption of innovative approaches which will ensure retention in care and optimal outcomes among HIV-infected children.

## Subjects and methods

### Study site

The study was conducted in NAUTH, Nnewi, Anambra State, Nigeria. The hospital offers tertiary services to the entire Anambra State and neighboring south-east Nigerian states. The paediatric ART programme formally started in the site in 2003. The NAUTH Paediatric HIV Unit is manned by adequately trained staff. Clinic consultations hold every working day (Mondays through Fridays) while in-patient services are offered on a daily basis.

Initiation of children on ART was based on Nigerian National HIV treatment guidelines which are in line with the World Health Organization (WHO) recommendations [8–11]. Prior to 2016, children were initiated on ART using eligibility criteria which included WHO clinical stage, immunologic (CD4 cell count) status and age of the child. However, Nigeria adopted the “test and treat” approach, in 2016, which recommended that ART should be initiated in all individuals with a diagnosis of HIV, regardless of WHO clinical stage and CD4+ cell count, within two weeks of diagnosis [11]. In addition to ART, the children were routinely screened for opportunistic or co-infections such as Tuberculosis, *Pneumocystis jirovecci* pneumonia, Hepatitis B and C among other infections. They also received prophylaxis for the opportunistic infections or treatments where necessary. All treatments were monitored clinically, and with laboratory investigations.

Generally, children on ART were seen at two-weeks after ART initiation, then monthly for three months. Thereafter, they were seen two-monthly, except when unstable. During the pre-ART era, visits for the children in the pre-ART pool was three monthly or less depending on their clinical condition. The children were routinely tracked to ensure continued engagement in care using standard guidelines, and procedures. HIV clinic appointments were scheduled in appointment registers. Children who missed scheduled appointments were daily identified and subsequently tracked through phone calls to their parents or caregivers.

### Methods

The records of all children enrolled into NAUTH paediatric ART programme over a 17 year period (1st January 2003 to 31st December 2019) was reviewed to document the trend in attrition Data of the children were prospectively recorded in the ART treatment registers domiciled in the clinic and on an electronic platform. This included their date of birth, date of enrolment, age and clinical parameters at enrolment, address, sex, and phone contact and caregiver details. On every clinic visit, the record was updated to reflect dates of subsequent visits, anti-retroviral drugs (ARV) status and regimen, as well as and the next appointment date. The date of cessation of care in the unit was also recorded together with final outcomes such as transfer to other sites, transition to adult ART programme, death or loss to follow-up. These data were abstracted using a proforma between 1st and 31st July 2020.

Using the phone numbers provided, caregivers of those who were lost to follow-up were contacted to determine further outcomes such as death, alive and receiving care elsewhere or alive but not in any form of care. Those who were alive but not in any form of care were encouraged to return to care. Details of all children who were alive but not in care and those who could not be reached were handed over to the Home Based Tracking Unit (HBTU) for further tracking and possible home visit.

### Data analysis

Data was analyzed using SPSS version 22. The primary outcomes were retention in care (still in Paediatric ART care, transitioned to adult ART or transferred out to another facility) or attrition (documented death or lost to follow-up). For the purpose of this study, loss to follow-up was defined as failure to return to care for ≥6 months after a scheduled clinic appointment. Retention in paediatric ART care was defined as being alive and receiving care from the unit, or documented transition to adult ART program or transfer to another site offering paediatric ART services. Death was defined as documented death within the period of care either while on admission in the facility or prior to a scheduled follow-up visit.

The duration of care or time to event was determined by subtracting the date of enrolment from the date of censoring. Those dead, lost to follow-up or transferred were censored on the date of their last hospital visit while those in care were censored on the final date of data abstraction (31st July 2020). The attrition rate per 1000 person years was calculated (using the formula attrition/1000PY = [number of attrition/cumulative person time years] × 1000) for the entire population as well as different categories.

The characteristics of children who were retained in care, dead or lost to follow-up were described using frequency tables for categorical variables while the continuous variables were described using median and interquartile range (IQR) or mean and standard deviation where applicable. The difference in these characteristics were compared using Pearson’s Chi-square test for categorical variables, Kruskal Wallis test for non-normally distributed continuous variables and one-way ANOVA for normally distributed continuous variables. Kaplan Meier curves with log rank tests were used to describe and compare attrition according to use of ARVs or not. Outcomes, overall attrition rate, and 6-, 12-, 24- and 36-months attrition rates were represented in charts according to year of enrolment. Cox regression analysis was used to examine the association between attrition and some socio-demographic factors. Significance level was set at 5%.

## Results

### Baseline characteristics

A total of 1213 children less than 15 years were referred to NAUTH Paediatric ART unit during the period under review. However, 99 (8.2%) children were excluded from the final analysis because they were never successfully engaged in care.

Final analysis for attrition or retention in care was done among 1114 HIV-positive children who were enrolled and followed-up in the Paediatric ART Programme of NAUTH, Nnewi. Some of their baseline characteristics are shown in Table 1. The male: female ratio was approximately 1:1. The age at enrolment ranged from one month to 14 years 11 months with a median and inter-quartile range (IQR) of 4.3 and 6.9 years, respectively. About a third (38.0%) of them belonged to age group 1 to < 5 years at enrolment. Majority (71.4%) did not reside within 15 km radius of the hospital and had one or both parent (64.5%) as caregiver. Year 2006 had the highest number of enrolments (13.6%). Approximately two-thirds (63.2%) of them had advanced HIV disease (WHO stage 3 or 4) at enrolment.

**Table 1:** Baseline characteristics of children enrolled into NAUTH Nnewi paediatric ART programme between 2003 and 2019

Characteristic (***n*** = 1114)	Frequency (%)
**Sex**	
Male	547 (49.1)
Female	567 (50.9)
**Age at enrolment**	
< I year	183 (16.4)
1 to < 5 years	423 (38.0)
5 to < 10 years	307 (27.6)
10 to < 15 years	201 (18.0)
**Clinical stage at enrolment**	
WHO Stage I or II	397 (35.7)
WHO stage III or IV	704 (63.2)
Not available	13 (1.2)
**Place of residence**	
Same state ≤15 km from site	318 (28.5)
Same state > 15 km from site	664 (59.6)
Other states	132 (11.8)
**Caregiver**	
Parent(s)	718 (64.5)
Elder sibling	201 (18.0)
Extended family member	13 (1.2)
Family friend	23 (2.1)
Institutional home	9 (0.8)
Not available	150 (13.5)
**Year of enrolment**	
**2003–2005**	142 (12.7)
**2006–2008**	394 (35.4)
**2009–2011**	317 (28.5)
**2012–2014**	137 (12.3)
**2015–2017**	76 (6.8)
**2018–2019**	48 (4.3)

### Outcomes

As shown in Table 2, the rate of loss to follow-up and death were 41.0 and 8.4% respectively giving a total attrition rate of 49.4%. Loss to follow-up accounted for majority (83.1%) of attrition.

**Table 2:** Outcomes of children enrolled into NAUTH Nnewi Paediatric ART Programme

Characteristics	Frequency (%)
**Outcome (n = 1114)**	
In care	226 (20.3)
Transitioned to adult ART programme	223 (20.0)
Transferred out to other paediatric ART sites	115 (10.3)_
LTFU	457 (41.0)
Dead	93 (8.4)
**Attrition rate (n = 1114)**	
In care, transitioned or transferred out	564 (50.6)
LTFU or dead	550 (49.4)
**Tracking outcomes of children LTFU (*n*** **= 457)**	
Self-referral to other ART sites	31 (6.8)
Alive and not in care	46 (10.1)
Dead while LTFU	43 (9.4)
Could not be reached	337 (73.7)
**Duration of care (n = 1114)**	
**< 1 year**	336 (30.1)
**1 to < 5 years**	323 (29.0)
**5 to < 10 years**	287 (25.8)
**10 to < 15 years**	164 (14.7)
**> =15 years**	4 (0.4)

*ART* anti-retroviral treatment, *LTFU* lost to follow-up

The final outcome of majority (73.7%) of children who were lost to follow-up could not be ascertained because phone contact was not provided in 56.7% (191/337) of them or the available phone contact could not be reached (either switched off or belonged to a stranger) in 43.3% (146/337) of them.

**Fig. 1:**
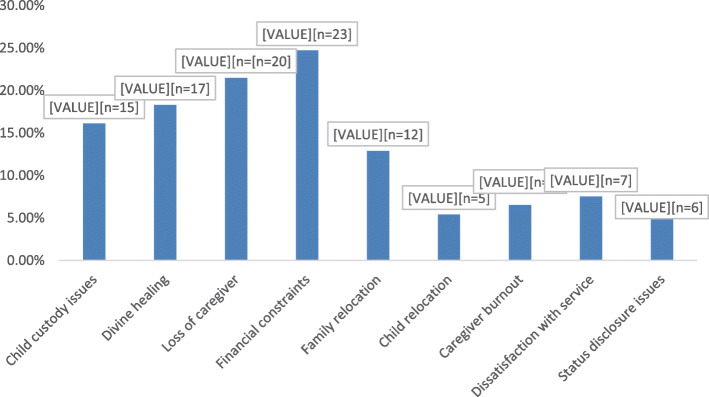
Reasons for LTFU (*n* = 93, multiple responses)

As shown in Fig. 1, the commonest reason cited for loss to follow-up among 93 out of 120 caregivers who were successfully contacted was financial constraints (including inability to meet up with cost of transportation and ancillary investigations). Other common reasons were loss of caregiver, claim to divine healing, family disharmony and child custody issues, and relocation of the family. Five children were relocated to another part of the country to serve as domestic helps to other families who were unaware of their HIV status. Twenty-eight (23.3%) of them did not proffer any reason for being lost to follow-up.

### Trend in attrition

**Fig. 2:**
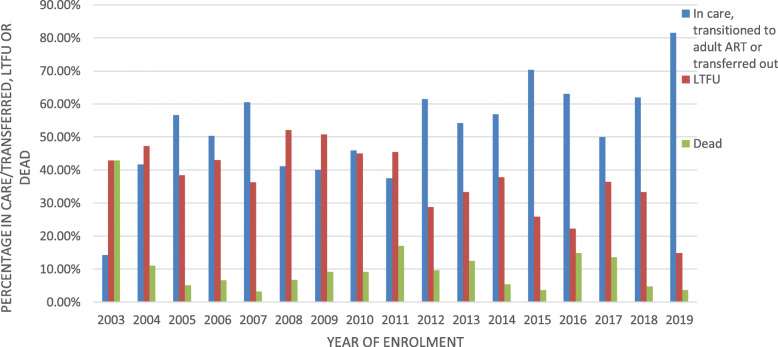
Trend in outcomes according to year of enrolment. *LTFU = Lost to follow-up*

Figure 2 shows an increase in the retention rate from 14.3 to 81.5% among those enrolled in 2003 and 2019, respectively. As shown in Table 3, the overall attrition rate decreased from 85.7 to 18.5% among children enrolled in 2003 and 2019, respectively. However, the decrease was not steady and fluctuated over the years. Attrition rate was higher than 50% among those enrolled between years 2003 to 2011, (apart from 2005, 2006 and 2007). A decrease below 50% occurred from 2012 to 2019 (apart from 2017). The 6-, 12-, 24- and 36-months attrition rates fluctuated over the years. The 6 months attrition was highest among children enrolled in 2017 (45.5%), 2003 (42.9%), 2011 (33.0%) and 2013 (29.2%). Similar trend was also noted in the 12-, 24- and 36-months attrition rates. Overall, the 6-, 12-, 24- and 36-months attrition rates were 20.4, 27.7, 34.3 and 37.3%, respectively as shown in Table 3.

**Table 3:** Trend in attrition rates according to year of enrolment

Enrolment Year (n)	Attrition rates according to year of enrolment				
	Six months	One year	Two years	Three years	Up to Dec. 2019
2003 (7)	3(42.9)	4(57.1)	5(71.4)	5(71.4)	6(85.7)
2004 (35)	8(22.2)	11(30.6)	14(38.9)	15(41.7)	21(58.3)
2005 (99)	13(13.1)	18(18.2)	21(21.2)	23(23.2)	43(43.4)
2006 (151)	34(22.5)	42(27.8)	49(32.5)	53(35.1)	75(49.7)
2007 (124)	22(17.7)	28(22.6)	35(28.2)	38(30.6)	49(39.5)
2008 (119)	20(16.8)	31(26.1)	34(28.6)	38(31.9)	70(58.8)
2009 (120)	27(22.5)	36(30.0)	46(38.3)	53(44.2)	72(60.0)
2010 (109)	18(16.5)	29(26.6)	42(38.5)	46(42.2)	59(54.1)
2011 (88)	29(33.0)	38(43.2)	46(52.3)	48(54.5)	55(62.5)
2012 (52)	8(15.4)	12(23.1)	13(25.0)	17(32.7)	20(38.5)
2013 (48)	14(29.2)	16(33.3)	18(37.5)	19(39.6)	22(45.8)
2014 (36)	6(16.2)	10(27.0)	14(37.8)	15(40.5)	16(43.2)
2015 (26)	4(14.8)	5(18.5)	8(29.6)	8(29.6)	8(29.6)
2016 (26)	4(14.8)	7(25.9)	9(33.3)	10(37.0)	10(37.0)
2017 (21)	10(45.5)	10(45.5)	11(50.0)	11(50.0)	11(50.0)
2018 (20)	4(19.0)	6(28.6)	7(38.9)	–	8(38.1)
2019 (26)	3(11.1)	3(16.7)	–	–	5(18.5)
**Total (1114)**	**227(20.4)**	**306(27.7)**	**372(34.3)**	**399(37.5)**	**550(49.4)**

### Characteristics of children who were dead, lost to follow-up or retained in care

As shown in Table 4, advanced disease (WHO stage 3 or 4) accounted for 58.2, 66.4 and 87.8% of children who were retained in care, lost to follow-up or died, respectively. More than half of children who were lost to follow-up (56.4%) or died (59.2%) did so within first year of care. Likewise, among those initiated on ARV, more than half of those who were lost to follow-up (55.8%) or died (55.7%) did so within first year of commencement on ARVs.

**Table 4:** Characteristics of children retained in care, dead or LTFU

Characteristics	In care or referred	LTFU	Dead	*p*-value
**Clinical stage at enrolment (*n*** **= 1101)**				
WHO stage 1	75(13.3)	44(9.9)	3(3.2)	
WHO stage 2	161(28.5)	105(23.6)	9(9.7)	
WHO stage 3	297(52.7)	247(55.6)	53(57.0)	
WHO stage 4	31(5.5)	48(10.4)	28(30.1)	
**Duration of care**	**(*n*** **= 564)**	**(n = 457)**	**(n = 93)**	
< 6 months	9(1.6)	192(42.0)	42(45.2)	
6 months to < 12 months	14(2.5)	66(14.4)	13(14.0)	
1 to < 5 years	165 (29.3)	137(30.0)	21(22.6)	
5 to < 10 years	227 (40.2)	48(10.5)	12(12.9)	
10 to < 15 years	145 (25.7)	14(3.1)	5(5.4)	
≥ 15 years	4 (0.7)	0 (0.0)	0 (0.0)	
Median care duration (IQR) in years	6.9(6.3)	0.7(2.8)	0.6(3.3)	< 0.001*
**Age at enrolment (years)**	**(n = 564)**	**(n = 457)**	**(n = 93)**	
< 1 year	79(14.0)	84(18.4)	20(21.5)	
1 to < 5 years	198(35.1)	189(41.4)	36(38.7)	
5 to < 10 years	163(28.9)	121(26.5)	23(24.7)	
10 to < 15 years	124(22.0)	63(13.8)	14(15.1)	
Median enrolment age (IQR) in years	5.1(7.7)	3.8(6.0)	2.6(6.3)	< 0.001*
**Age at last visit (years)**	**(n = 564)**	**(n = 457)**	**(n = 93)**	
< 1 year	2(0.4)	43(9.4)	10(10.8)	
1 to < 5 years	33(5.9)	152(33.3)	30(32.3)	
5 < 10 years	104(18.4)	139(30.4)	25(26.9)	
10 to < 15 years	201(35.6)	105(23.0)	26(28.0)	
> =15 years	224(39.7)	18(3.9)	2(2.2)	
Median last visit age (IQR) in years	14.1(5.4)	6.0(7.7)	6.3(9.4)	< 0.001^*^
**Use of ARV**				
Yes	538 (95.4)	258(56.5)	70(75.3)	
No	26(4.6)	199(43.5)	23(24.7)	
**Enrolment and ARV initiation interval (months)**	***n*** **= 538**	***n*** **= 258**		
Mean ± SD	12.9 ± 25.84	6.7 ± 17.45	7.8 ± 16.51	< 0.001^†^
**ARV initiation and last visit interval (years)**	**n = 538**	**n = 258**	***n*** **= 70**	
< 1 year	40(7.4)	144(55.8)	39(55.7)	
1 to < 5 years	197(36.6)	72(27.9)	20(28.6)	
5 to < 10 years	199(37.0)	33(12.8)	7(10.0)	
10 years< 15 years	102 (19.0)	9 (3.5)	4 (5.7)	
Median ARV initiation and last visit interval (IQR)	5.7(6.1)	0.6(3.0)	0.6(2.8)	< 0.001^*^

*LTFU* loss to follow-up, *IQR* interquartile range, *SD* standard deviation, *ARV* anti-retrovirals

^*^Statistically significant Kruskal-Wallis test, ^*†*^*Statistically significant one-way ANOVA*

A higher proportion of those who were lost to follow-up or died were aged 1 to 5 years at enrolment or last hospital visit. The highest number of loss to follow-up occurred between 2009 and 2011 (10.1%). On the other hand, the highest number of deaths occurred in 2011 (20.4%) and 2012(18.3%). Majority of the children who died were commenced on ARVs (75.3%) while more than half (56.5%) of those who were lost to follow-up were on ARVs.

**Fig. 3:**
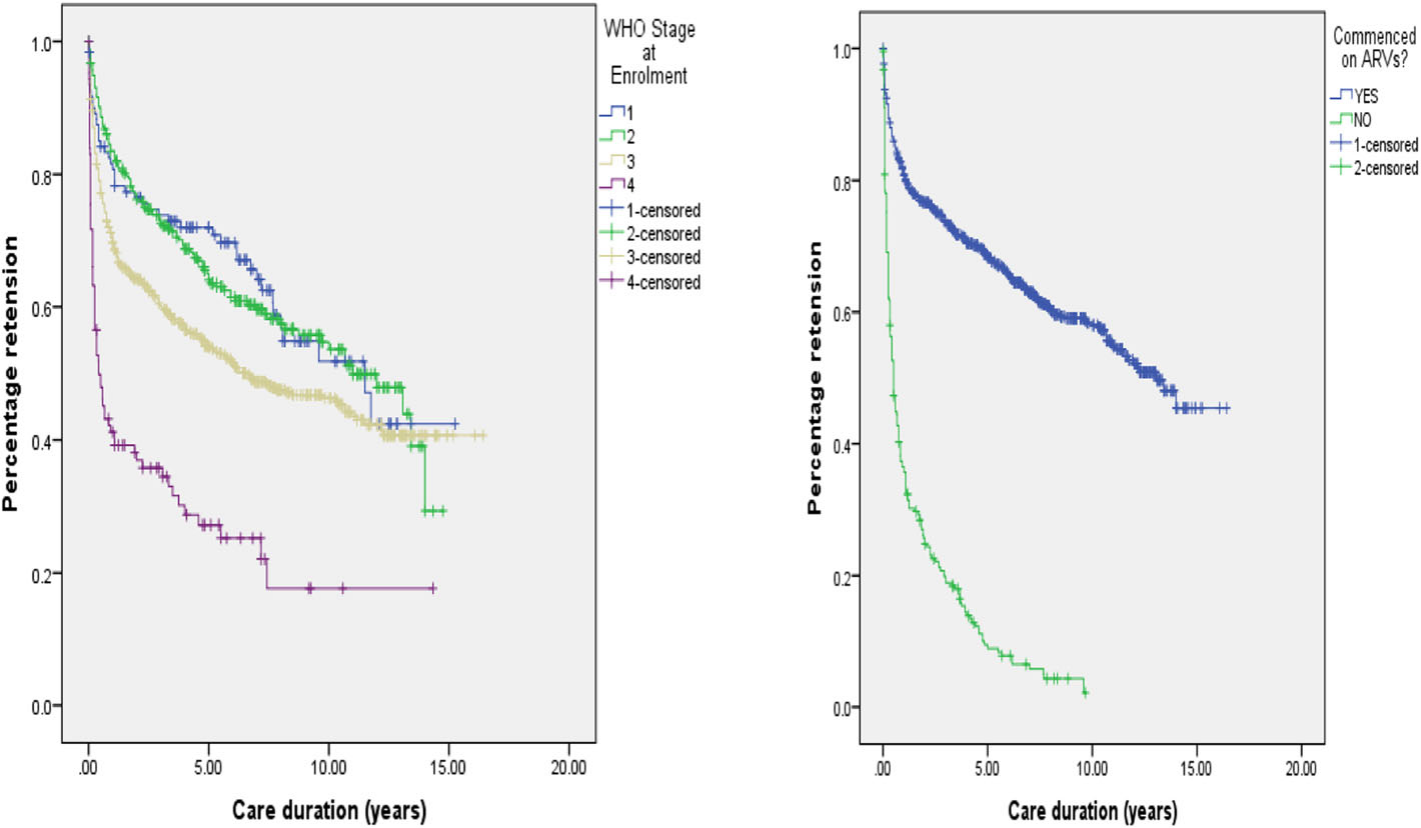
Kaplan-Meier survival curves according to initial WHO clinical stage or use of ARVs

The duration of care, age at enrolment or last hospital visit and interval between ARV initiation and last hospital visit were comparable between children who died or were lost to follow-up. Children who were retained in care had a longer duration of care, and interval between ARV initiation and last hospital visit. A higher proportion of children retained in care belonged to an older age at enrolment. As shown in Fig. 3, retention of children who had WHO stage 4 disease at enrolment as well as those who were not initiated on ARV rapidly decreased within the first year of care.

### Results of bivariate and multivariate analysis

As shown in Table 5, the overall incidence in attrition/1000 person years was 108. Children who had advanced disease at enrolment, were not initiated on ARV or less than one year of age at enrolment had a higher incidence in attrition/1000PY. Likewise, those who lived outside Anambra state, whose primary caregiver was a non-family member, and in descending order, those who enrolled in 2018–2019, 2009–2011 and 2015–2017 had higher incidence of attrition/1000 person years. Time to attrition was significantly lower among those who had advanced disease at enrolment or were not commenced on ARV, as well as children enrolled between 2015 and 2017 or 2009–2014. Age less than one year at enrolment, having non-family member as primary caregiver and living outside Anambra state had the least time to attrition but these did not achieve statistical significance.

**Table 5:** Result of bivariate and multivariate analysis of factors associated with attrition

Variable	Total	PT Years	Attrition (%)	*P*-value	Estimated timeto event (years)	*p*-value	Attrition/1000PY	B	HR	*P*-value
Total	1114	5109.7	550(49.4)		7.3		108			
Clinical stage										< 0.001^c^
WHO stage 1	122	646.1	47(38.5)		11.5	< 0.001	73	−1.81	0.16(0.11–0.25)	< 0.001
WHO stage 2	275	1488.2	114(41.5)	< 0.001^a^	11.0		77	−1.62	0.20(0.14–0.28)	< 0.001
WHO stage 3	597	2767.0	300(50.3)		6.7		108	−0.96	0.39(0.29–0.52)	< 0.001
WHO stage 4	107	203.8	76(71.0)		0.4		373	–	Reference	–
**Use of ARVs**										
Yes	866	4762.9	328(37.9)	< 0.001^a^	13.1	< 0.001^b^	69	−2.13	0.12(0.09–0.15)	< 0.001^c^
No	248	346.8	222(89.5)		0.5		640	–	Reference	
**Sex**										0.873
Male	547	2603.9	266(48.6)	0.626	7.4	0.375	102	5.09	161.99	0.859
Female	567	2505.8	284(50.1)		6.7		113	5.04	154.27	0.860
**Enrolment Age (years)**										0.001^c^
< 1	183	874.8	104(56.8)		5.3		568	0.72	2.05 (1.40–3.00)	< 0.001^c^
1 to < 5	423	2205.2	225(53.2)	0.001^a^	6.9	0.121	102	0.35	1.41 (1.02–1.96)	0.037
5- < 10	307	1371.9	144(46.9)		6.8		105	0.16	1.17(0.83–1.66)	0.362
10 to < 15	201	657.8	77(38.3)		7.3		117	–	Reference	–
**Caregiver**										0.142
Parent(s)	718	3721.1	307(42.8)		11.5		83	−0.21	0.81 (0.49–1.34)	0.417
Sibling	13	46.8	1(7.7)		–		21	−1.65	0.19(0.03–1.45)	0.109
Extended family member	201	910.6	95(47.3)	0.026^a^	7.8	0.073	104	−0.01	0.99(0.59–1.68)	0.981
Others	32	138.5	17(53.1)		5.1		123	–	Reference	–
**Place of residence**										0.577
Same state ≤15 km radius	162	810.9	79(48.8)	0.058	8.1	0.065	97	−0.21	0.81(0.49–1.34)	0.299
Same state > 15 km from site	820	3839.7	400(48.8)		7.5		104	−0.11	0.90(0.65–1.24)	0.515
Other states	132	459.1	71(53.8)		3.9		155	–	Reference	–
**Period of enrolment**										< 0.001^c^
2003–2005	142	889.2	70(49.3)		9.2		79	−0.25	0.78(0.38–1.63)	0.512
2006–2008	394	2300.7	194(49.2)		8.3	0.001^b^	84	−0.67	0.51(0.25–1.04)	0.063
2009–2011	317	1171.7	186(58.7)	< 0.001^a^	5.5		159	−0.19	0.83(0.41–1.67)	0.595
2012–2014	137	499.8	58(42.3)		5.2		116	−0.05	0.95(0.46–1.97)	0.897
2015–2017	76	191.4	29(38.2)		3.5		152	−0.00	1.00(0.46–2.20)	0.997
2018–2019	48	57.0	13(27.1)		2.0		228	–	Reference	–

^*a*^*Statistically significant chi-square test* ^*b*^*Statistically significant Log rank (Kaplan Meier)*

^*c*^*Statistically significant Cox regression B = regression coefficient HR = Hazard ratio*

In bivariate analysis, WHO clinical stage and age at enrolment, not being commenced on ARV, primary caregiver, and period of enrolment were associated with attrition. Apart from primary caregiver, these factors remained significant in multivariate analysis. Use of ARVs reduced the risk of attrition by 88%. Enrolment age less than one year, and one to five years had 105 and 41% higher risk of attrition, respectively, compared to age 10 years or above. Living within 15 km to the hospital reduced attrition by 19% compared to those who lived outside Anambra State, although this was not statistically significant. Having elder sibling or parent(s) as primary care giver reduced attrition by 81 and 19%, respectively, compared to a non-family member although this factor did not attain statistical significance in multivariate analysis.

## Discussion

Our findings portray a high attrition rate among children living with HIV. Among children who were referred to the unit, 8.2% did not complete the enrolment process and return for the next visit. Among those who were successfully linked to care, the overall attrition rate was 49.4% with incidence of 108/1000 person years while the 6-, 12-, 24-, and 36-months attrition rates were 20.4, 27.7, 34.3 and 37.5%, respectively. These findings are comparable to reports from similar African studies [12–19].

The pre-engagement attrition highlights the success of linkage to care. Successful linkage to care requires navigation through post-test counselling, transfer to clinic, enrolment protocols, initial clinical and laboratory evaluation, ART initiation and first follow-up visit. Some of these services may be hindered by health system related factors such as user fees and long waiting time. In addition, as revealed by our findings, many children present with advanced HIV disease and may die before the first follow-up visit. Children are completely dependent on adult caregivers to access care. Therefore, caregiver issues such as status denial, fear of stigmatization, ill health, death and financial constraints could hinder successful linkage to care. Current efforts to address the gap of successful linkage include rapid ART initiation and case management approach [1–23]. The case management approach ensures that a newly diagnosed HIV positive individual is successfully linked to coordinated health and social services to achieve desired outcomes [24]. This approach is patient centered, an ongoing rather than a one-off process, and responds to the peculiar needs of the client [24]. Although these interventions have been demonstrated to improve successful linkage to care, their implementation should be intensified and monitored to achieve desired results.

The findings of this study are in keeping with previous reports of high attrition rates in West African Paediatric ART Programmes [15, 18, 25]. The West African sub-region has been reported to have the poorest retention in care compared to other parts of the world. In addition, a wide disparity exists between attrition rates in developed and developing countries [26, 27]. Urgent innovative interventions are needed to address the high attrition rates in West Africa in order to achieve the 2030 global targets [7]. Reports consistently show that loss to follow-up constitute majority of attrition among children enrolled in HIV treatment programmes in developing countries [12–19, 27, 28]. However, this should be interpreted with caution because a significant proportion of loss to-follow up may be actual deaths. This hypothesis was supported by the finding that children who were lost to follow-up shared similar characteristics with those who died and this is substantiated by previous report [29]. The fact that majority of children who were lost to follow-up could not be reached on phone was somewhat disappointing. The inability to reach them could be attributed to loss/replacement of sim cards as well as the multiplicity of individual sim card ownership in the country. This challenge buttresses the need for innovative tracking approach that will ensure efficient real-time tracking, quick re-engagement in care and proper documentation of true outcomes [30]. Adequate funds should be earmarked for tracking down to the household and community levels should phone calls fail as experienced in the index study.

The findings from caregivers who were successfully reached provided insight into the outcomes of children who were lost to follow-up. More than a third died while lost to follow-up (35.3%) or were alive but not in any form of care (38.7%), while a quarter (26.1%) started accessing care at other sites. This reinforces the need for innovative strategies that can identify enrollees who were previously in care or ART experienced to ensure appropriate documentation and reporting of outcomes. The reasons for being lost to follow-up highlight the challenges faced by HIV-infected children and their caregivers. This is buttressed by a Zimbabwean report [31]. Expectedly, most of the issues were caregiver related. Our findings agree with a report which showed that financial constraints pose the commonest barrier to continued engagement in care in developing countries [25]. Although anti-retroviral drugs are free, families may not meet up with the cost of certain services including ancillary laboratory investigations, folder/card fees, treatment of co-morbidities, as well as frequent transportation to and from hospitals. More so, this may be aggravated by the indirect cost of the lifelong sickness on the family. In order to address these challenges, the World Health Organization adopted a public health approach in HIV programmes. This includes task sharing, decentralization and integration of HIV services with other health services and differentiated service delivery (DSD). Task shifting and decentralization of services to peripheral health centers are believed to increase the number of healthcare workers and facilities providing ART services, thereby, bringing services closer to clients’ doorsteps. However, accessing care from a nearby facility may not be acceptable to caregivers due to fear of stigmatization. Therefore, efforts to minimize stigmatization should be intensified to ensure that children are treated in facilities as close as possible to their homes in order to reduce transportation cost.

The differentiated service delivery is a client centered approach that focuses on the preferences, needs and values of clients while reducing unnecessary burden on the healthcare facilities. However, children were not prioritized in the initial scale up of DSD. This was attributed to the fact that children often have age-related clinical issues that may require specialist care or more intensive follow up. For instance, rapid growth during the first two years of life necessitates frequent weight monitoring and weight-based dosage adjustment. However, children above 2 years can benefit from multi month scripting, and family-centered approaches including same-day appointments, duration of ART refills or allowing family member to collect ART refills. DSD has been documented to optimize quality of care, efficiency, client satisfaction and continued engagement in care. Therefore, the application of some DSD models should be explored in paediatric ART programmes. In addition, programmes should adopt child-centered interventions that can step in when HIV-infected children experience complex problems, which have the potential to terminate their treatment. Such interventions may include legal support especially for children in unstable homes, enforcement of legislation against child labour, and economic empowerment for the child’s family. Efforts should be made to ensure that HIV-positive children remain on treatment irrespective of caregiver’s religious belief, death, relocation and financial status. Quality adherence counselling and psychosocial support for the child and family are vital to the success of paediatric ART programmes.

Unexpectedly, the trend in attrition failed to assume a steady decline despite the reported scale up of case identification, linkage to care and treatment monitoring. This may reflect fluctuation in programme funding and treatment policies [1, 5]. Reports consistently show an association between delay in initiation on ART or very young age and high risk of attrition [12, 13, 15, 25, 32–35]. This justifies the adoption of the “test and treat” and rapid ART initiation approaches [24]. However, the patients should be closely monitoring during the first 6 months of ART since this period has been consistently reported to account for a significant proportion of attrition [12, 13, 15, 25–27, 32–35]. The under-fives represent a highly vulnerable population due to rapid disease progression and high risk of death. Hence, this age group should be closely monitored to forestall death or loss to follow-up. Furthermore, programmes should ensure potent age-appropriate regimens and dosages. Our finding that about two-thirds of all children had advanced WHO clinical stage at enrolment agrees with previous African reports [ 13, 17, 25–31]. This calls for intensified efforts at identifying infected pregnant women and linking them to prevention of MTCT programmes as well as early infant diagnosis.

The association between attrition and having parent(s) as caregiver agrees with previous reports [13, 31]. This may not be unconnected with the fact that the parent(s) may be sick or die eventually, may be in denial of status, or experiencing financial difficulties due to ill health. These may explain the high attrition also recorded in adult ART programmes [36, 37]. Therefore, parents need adequate support to enable them remain alive and healthy, and to continue to take care for their infected children. Surprisingly, only one out of 13 children who had elder sibling as caregiver died and none was lost to follow-up. Programmes can explore the possibility of engaging elder siblings as secondary caregivers or primary caregivers in the event of loss of parent(s), rather than transferring care to other family members or non-family members thereby increasing the risk of attrition.

## Conclusion and recommendations

The Paediatric HIV treatment programme in Nnewi, Nigeria recorded a high rate of attrition which was mostly due to loss to follow-up. Most children lost to follow-up could not be reached on phone. Major reasons for being lost to follow-up were financial constraints, loss of caregiver, claim to divine healing, family disharmony, child custody issues, and relocation of family or child. Predictors of attrition were advanced HIV disease, young age at enrolment, delay in initiating ARVs, primary caregiver, and period of enrolment.

HIV treatment service delivery should be tailored to the needs of the child and his/her family. Efforts should be intensified at early diagnosis, successful linkage to care and effective implementation of the “test and treat” strategy. Innovative child centered approaches and interventions should be adopted to enable the HIV-infected child continue treatment despite challenges which have the potential to truncate treatment. Removal of user fees and re-activation of home-visit as a component of tracking are recommended.

## Data Availability

The study dataset is available and can be obtained from the corresponding author on reasonable request.
